# Optimizing the Use of SARS-CoV-2 Antigen Rapid Diagnostic Tests for the Timely Detection of and Response to COVID-19 in Schools and Markets in Uganda

**DOI:** 10.4269/ajtmh.23-0758

**Published:** 2024-11-26

**Authors:** Jerry Mulondo, Susan Nayiga, Winnie Nuwagaba, Patience Nayebare, Jane Frances Namuganga, Isaac Ssewanyana, Moses R. Kamya, Joaniter I. Nankabirwa

**Affiliations:** ^1^ Infectious Diseases Research Collaboration, Kampala, Uganda;; ^2^ Department of Medicine, Makerere University College of Health Sciences, Kampala, Uganda

## Abstract

The early detection and management of infections is crucial to control epidemics. We evaluated the feasibility and utility of severe acute respiratory syndrome coronavirus 2 (SARS-CoV-2) antigen rapid diagnostic tests (Ag-RDTs) for the timely detection of and response to coronavirus disease 2019 in high-risk border communities in Uganda. Between May and September 2022, monthly cross-sectional surveys were conducted in 11 schools and two markets in two border districts. Only baseline and end-line testing were also performed in matched control communities. Antigen rapid diagnostic test results and demographic and clinical data were collected, and contacts of patients were traced and tested. All patients were advised to self-isolate, and compliance was assessed on day 5. We enrolled 10,406 participants out of 10,472 screened individuals. The participants had a 1.3% test positivity rate, with schools recording higher, but non-significant, positivity rates than markets (1.4% versus 0.9%; *P* = 0.149). We tracked 556 contacts, and 536 (96.4%) agreed to test. The test positivity rate was significantly higher among contacts than the index participants (8.8% versus 1.3%; *P* <0.001). Only 55 (29.7%) of the index participants self-isolated effectively. Settings that received monthly testing had lower end-line positivity rates than controls (0.3% versus 1.4%; *P* = 0.001). Repeated SARS-CoV-2 Ag-RDT testing is feasible and could reduce SARS-CoV-2 infections. However, the participation in testing may have been enhanced by the compensation provided. Also, isolation was limited, which may reduce the impact of the intervention when rolled out on a large scale. Innovative strategies to increase the isolation of patients could improve the utility of early testing for transmission reduction during epidemics.

## INTRODUCTION

Severe acute respiratory syndrome coronavirus 2 (SARS-CoV-2), which causes coronavirus disease 2019 (COVID-19), has been associated with substantial morbidity, mortality, and disrupted social and economic structures.^1^ In Uganda, the SARS-CoV-2 pandemic resulted in significant mortality^2^^,^^3^ and overwhelming hospitalizations, with shortages of drugs, hospital beds, health workers, and acute intensive care services.^4^^,^^5^ This impacted healthcare-seeking behavior, case management, and the delivery of preventive measures^4^^,^^6^^,^^7^ and led to disruptions in schooling with increased cases of child abuse, teenage pregnancies, and community violence.^8^^–^^12^ Similar to other respiratory infections, the transmission of SARS-CoV-2 infections is highest in places where people congregate (such as schools and markets) because it is hard to observe prevention guidelines in such settings.^13^^–^^16^ Transmission is worsened when these communities are located in border districts, where people are highly mobile and travel between different geographical regions is not controlled, promoting the spread of new infections from imported cases.^17^ These settings may benefit most from the early detection and management of positive cases to reduce transmission, especially during epidemics.

SARS-CoV-2 antigen rapid diagnostic tests (Ag-RDTs) are a valuable tool to extend repeated testing into high-risk communities because they are relatively inexpensive, simple to perform, do not require a specialized laboratory, and have a short turnaround time (15–30 minutes). As such, Ugandan SARS-CoV-2 Ag-RDTs are recommended for community testing.^18^ Unfortunately, their use to inform decisions, such as reopening schools, returning to work, mass gatherings, and travel, was limited. The initial consequences of this limitation were delays in case management, isolation, and the easing of restrictions imposed to curb the spread of SARS-CoV-2. With the control of the epidemic, SARS-CoV-2 Ag-RDT testing at the population level may still be essential for surveillance. In this study, we conducted operational research in schools and markets in the border districts of Tororo and Busia in Uganda with the aim of demonstrating the feasibility and utility of Ag-RDTs as a tool for repeated screening for SARS-CoV-2 infections in high-risk communities.

## MATERIALS AND METHODS

### Study design.

Between May and September 2022, serial monthly cross-sectional surveys were conducted in a random sample of 11 schools and two markets in the border districts of Busia and Tororo, Uganda. As part of the surveys, a brief questionnaire adopted from the Uganda Ministry of Health (MoH) COVID-19 data collection tool was used to collect information on demographics, clinical characteristics, and SARS-CoV-2 Ag-RDT testing and conducted on all participants. The contacts of the participants with positive test results were identified and tested. All participants with positive test results were encouraged to self-isolate according to MoH guidelines, and follow-up calls were conducted on days 1, 2, 3, 4, and 5 post-diagnosis to determine the number of participants who successfully self-isolated. To estimate the number of infections averted, each selected school or market was matched with another school or market with similar characteristics and within the same geographical setting (control schools and markets). In the control schools and markets, testing was only conducted during the baseline and end-line surveys.

### Study setting.

The study was conducted in schools and markets located in the Tororo and Busia Districts of Uganda. The two districts were purposely selected because of their geographical location and economic activities. Both Tororo and Busia are located on Uganda’s border with Kenya and are the connections to the Mombasa Seaport, making them some of the busiest points of entry in the country. The two districts also host several resting places for long-distance truck drivers. This geographical location, together with the economic interactions, puts these districts’ populations at a higher risk of acquiring and transmitting emerging infections, including SARS-CoV-2 variants, than other districts in Uganda.^17^ The response to epidemics in both districts is led by the District Task Force, with technical guidance and support from the MoH and several developmental partners.

### Study population.

Before the study, sensitization meetings were held with the leadership and community advisory boards of the two districts. Lists of all schools and markets within the districts were obtained from the districts to provide a sampling frame for the studies. Schools on the list were stratified by level (primary, secondary, or tertiary school) and type (day or boarding school), and markets were stratified by ownership (government versus private). Using the random function in Microsoft Excel (Microsoft Corp, Seattle, WA), a random sample of schools and markets was selected from each stratum to participate in the study. In total, 11 schools and two markets were selected as part of this process. Each selected school or market was matched with another school or market with similar characteristics in terms of level (primary, secondary, or tertiary institution), type (day versus boarding; mixed, girls only, or boys only school; primary, secondary, or tertiary institution), ownership (government or privately sponsored school), and total number of students enrolled. For the markets, matching was performed on ownership and size.

At baseline, information was collected on all schools and markets in Tororo and Busia Districts. Information collected on the schools included 1) type of school (day versus boarding; mixed, girls only or boys only school; primary, secondary, or tertiary institution; government or privately sponsored school); 2) total enrollment; and 3) location of the school. Schools were matched on all parameters, including the type of school, total enrollment, and location. In each district, we selected one school to represent each type of school (primary, secondary, and tertiary in each district), and given the unique nature of the boarding versus day schools, we had a representative of each of the three levels for the day and boarding schools (12 schools per arm). However, all the tertiary institutions had a mix of day and boarding arrangements, and they were not stratified by day and boarding, thus resulting in 11 versus 12 per arm.

After the selection of participating schools and markets, sensitization meetings were held in the selected communities. Parents and caregivers of students in the selected schools were invited to take part in these sensitization meetings. At the end of these meetings, informed consent was sought from parents and caregivers of children under 18 years of age. On the survey days, participants were invited to participate in the study. Participants were screened for eligibility to join the study and were enrolled if they fulfilled the following criteria: 1) are residents of the selected communities; 2) have provided consent to participate in the study (parental consent for children); and 3) provided assent for children 8–17 years of age. Study participation in schools focused on school children, teaching staff, and support staff, whereas that in the markets focused on both the market vendors and the customers. Participants were enrolled depending on their availability at the time of testing, regardless of whether they had been tested previously or not, until the required sample size was attained. The subjects were compensated for participating in the study. The adults received UgSh10,000/= compensation for their time, whereas students received a math set or schoolbooks (all compensation was ∼$3). The study was approved by the Makerere University College of Health Sciences, School of Medicine Research and Ethics Committee (Mak-SOMREC-2022-295), and the Uganda National Council for Science and Technology (HS2050ES).

### Survey procedures.

A questionnaire adapted from the MoH COVID-19 data capture tools was administered to all participants to capture information on their demographics, presence of any COVID-19-related symptoms, COVID-19 vaccination status, and history of contact with a known COVID-19 case. To enhance privacy, the study procedures were conducted in designated rooms provided by the leadership of the school and markets. At the time of the survey, five vaccines were approved for use in Uganda, namely, Janssen vaccine (single dose, 0.5 mL, ≤ 0.15 *µ*g), AstraZeneca (two doses, 0.5 mL each, eight to twelve weeks apart), Pfizer (two doses, 30 *µ*g/0.3 mL each, four to eight weeks apart), Moderna (two doses, 100 *µ*g/0.5 mL each, one month apart), Sinopharm (two doses, 0.5 mL, two to four weeks apart), and Sinovac (two doses, 0.5 mL each, two to four weeks apart).

Nasal swab samples were collected by the research assistant from all participants for the SARS-CoV-2 Ag-RDT testing. All testing was conducted by using the Abbott Panbio^TM^ COVID-19 Ag kits (model 41FK10; Abbott Laboratories, Abbott Park, IL) according to the manufacturer’s guidelines. Tests were performed by study personnel, and the results were available within 15 minutes. The results of the SARS-CoV-2 Ag-RDT were provided to the participants verbally and were recorded in the participants’ forms.

When a volunteer tested positive on the rapid diagnostic test (index case), additional information was collected, including details on how to locate their homes and contacts. Contacts for school participants included household members, classmates, children sharing their dormitory, and playmates. Contacts for participants in markets included household members and workmates. All contacts of identified index participants were tracked by the study team and tested for the SARS-CoV-2 antigen by using a similar methodology to that of the participants. All participants (index and contacts) were provided with information on the home-based management of COVID-19 infections and advised to self-isolate according to national guidelines.^19^^,^^20^ Symptomatic participants were referred to the nearest health facility for further management. All participants were followed up by phone if they had one or with a home or school visit on days 1, 2, 3, 4, and 5 to assess for any signs or symptoms of disease progression and assess for adherence to the isolation recommendations.

### Data management.

All data were collected by survey teams using hand-held tablet computers. These contained information from the questionnaires and fields for entering results of the SARS-CoV-2 Ag-RDTs. Quality control checks were programmed into the devices and fully validated. All data were checked for completeness and accuracy before leaving the survey sites. Data from the tablet computers were transferred at the end of every day to our data core facilities in Kampala and stored on a secure server. The data file was kept on a separate network so that only authorized survey staff would have access to the data during the collection and processing phase.

All study personnel were trained in the study protocol and associated procedures.

## STATISTICAL ANALYSES

The sample size was estimated by using the formula for a single proportion. We hypothesized that at least 90% of the participants would have a test successfully performed. The sample size to test this hypothesis was estimated by using the formula for a single proportion and a 95% significance level and fixing the margin of maximum error at 5%. We needed to test at least 138 participants in each of the survey populations to meet this objective.

Data analysis was performed by using STATA, version 14 (Stata Corporation, College Station, TX). Feasibility was estimated by using the following two outcomes: 1) the proportion of participants for whom a test was successfully completed, which was defined as the proportion of participants and their contacts for whom a test is performed divided by the total number of participants identified (volunteering and contacts) for testing, and 2) the proportion of participants with positive test results who appropriately isolated. This was defined as the number of participants with positive test results (both index and contacts) who effectively self-isolated for at least 5 days divided by the total number of participants. The utility of using the SARS-CoV-2 Ag-RDT for the early detection of infections is defined by the following two outcomes: 1) Ag-RDT test positivity rates among the participants, which was defined as the number of participants testing positive by the SARS-CoV-2 Ag-RDT divided by the total number of participants tested; 2) factors associated with test positivity rates; and 3) the number of infections averted by regular testing, which was calculated as the comparison of the end-line test positivity rates among those receiving regular or monthly testing compared with the end-line test positivity rates of the controls.

Descriptive characteristics are summarized as proportions for categorical variables and median (interquartile ranges) for continuous variables. Age is categorized into three groups (under 12 years, 12–18 years, and above >18 years) on the basis of age ranges of school-aged children in primary schools and secondary schools and adults. Log-binomial regression models with generalized estimating equations were used to compare the test positivity rates for the different predictor variables and expressed as the prevalence ratio with their 95% confidence intervals. A *P*-value of <0.05 was considered statistically significant.

## RESULTS

### Feasibility of the study.

**Figure 1.**
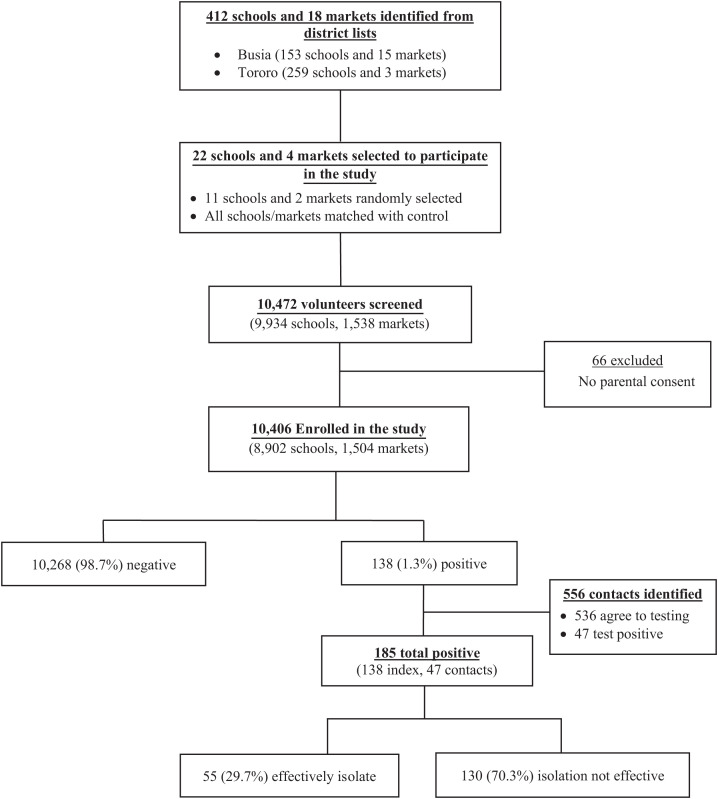
Flow of participants into the study.

The measure of feasibility was the ability to engage the leadership of the participating communities, incorporate the intervention within the communities, test at least 90% of the people who volunteer for testing, and be able to track and test the contacts of the participants who tested positive. In this study, a total of 412 schools and 18 markets were identified in the two districts, of which 11 schools and two markets were randomly selected to participate. Selected schools were matched with control schools or markets (giving a total of 22 schools and four markets). In the four surveys conducted, a total of 10,472 participants were screened, of whom 10,406 (99.4%) were enrolled in the study and all were successfully tested for SARS-CoV-2 infection (Figure 1). For 66 participants who were not enrolled, parental consent could not be obtained, and these participants were excluded from the study. In total, 556 contacts of the index participants were identified from the interviews with participants, and all were successfully tracked by the study team; however, only 536 (96.4%) consented to the SARS-CoV-2 Ag-RDT testing and were included in the study. We predetermined the feasibility estimate as being able to successfully test 90% of the participants. In this study, 99.4% of the participants and 96.4% of the contacts were successfully tested.

### Characteristics of the study population.

Overall, 10,942 subjects were enrolled in this study, including 10,406 participants and 536 contacts. Of these, 7,332 (67.1%) were enrolled from the monthly or regular testing communities and 3,610 (32.9%) were from the control communities. The median age of the participants was 18 years, and their ages ranged from 4 to 87 years. There were almost equal proportions of the male and female participants enrolled. The majority of the participants were schoolchildren.

The demographic and clinical characteristics of the study participants were comparable between the two communities (Table 1). Slightly more participants were enrolled from Tororo compared with Busia District (55.1% versus 44.9%), and most of the enrollments were from the surveys conducted in schools (9,397; 85.9%). Many of the participants had received at least one dose of the COVID-19 vaccine (7,042; 64.4%), although only 2,423 (22.2%) had completed the recommended doses. In total, 2,050 (18.7%) participants reported having symptoms related to COVID-19, with a runny nose being the most reported symptom. A few participants (171 [1.6%]) reported having had contact with a suspected COVID-19 patient within the last 2 weeks. During the baseline, only 6/3,413 (0.2%) participants tested positive for COVID-19 through the SARS-CoV-2 Ag-RDT, with equal numbers and proportions between the regular or monthly testing communities and the control communities (0.17% versus 0.18%; *P* = 0.944).

**Table 1:** Demographic and clinical characteristics of the participants enrolled in the study

Characteristics	Controls (*n* = 3,610)	Regular/Monthly Testing (*n* = 7,332)	Overall (*N* = 10,942)
Median age, years (IQR)*	18 (15–23)	18 (15–22)	18 (15–22)
Age categories (years)*			
<12	356 (10.1)	608 (8.8)	964 (9.3)
12–18	1,409 (40.1)	3,067 (44.5)	4,476 (43.0)
>18	1,748 (49.8)	3,218 (46.7)	4,966 (47.7)
Sex, *n* (%)*			
Male	1,973 (56.2)	3,358 (48.7)	5,331 (51.2)
Female	1,540 (43.8)	3,535 (51.3)	5,075 (48.8)
District, *n* (%)			
Busia	1,651 (45.7)	3,267 (44.6)	4,198 (44.9)
Tororo	1,959 (54.3)	4,065 (55.4)	6,024 (55.1)
Testing setting, *n* (%)			
School	3,110 (86.1)	6,287 (85.7)	9,397 (85.9)
Market	500 (13.9)	1,045 (14.3)	1,545 (14.1)
Vaccination status, *n* (%)			
None	1,128 (31.3)	2,772 (37.8)	3,900 (35.6)
Only first dose	1,677 (46.5)	2,942 (40.1)	4,619 (42.2)
Fully vaccinated	767 (21.2)	1,553 (21.2)	2,320 (21.2)
Received booster doses	38 (1.0)	65 (0.9)	103 (1.0)
Any COVID-19 symptoms, *n* (%)			
No	2,973 (82.4)	5,919 (80.7)	8,892 (81.3)
Yes	637 (17.6)	1,413 (19.3)	2,050 (18.7)
Any chronic medical conditions, *n* (%)			
No	3,502 (97.0)	7,162 (97.7)	10,664 (97.5)
Yes	108 (3.0)	170 (2.3)	278 (2.5)
Contact with suspect in last 2 weeks, *n* (%)			
No	3,566 (98.8)	7,205 (98.3)	10,771 (98.4)
Yes	44 (1.2)	127 (1.7)	171 (1.6)
Baseline RDT results^†^			
Negative	1,658 (99.83)	1,747 (99.82)	3,407 (99.82)
Positive	3 (0.18)	3 (0.17)	6 (0.18)

COVID-19 = coronavirus disease 2019; IQR = interquartile range.

* Data only available for participants.

^†^ Only baseline survey results included.

### Test positivity rates and isolation rates.

The total number of positive patients identified was 185/10,942, giving an overall test positivity rate of 1.7% (Table 2). The test positivity rate was significantly lower among the participants compared with the contacts (1.3% versus 8.8% *P* <0.001). Of the 185 participants who tested positive using the Ag-RDT, 120 (64.9%) had COVID-19-related symptoms, and of those with symptoms, 82 (68.3%) had sought health care from a public health facility for their symptoms in the last week. However, none of the participants who had sought care had a COVID-19 test performed. Of the 185 patients identified, only 55 (29.7%) followed the recommendations on self-isolation. The proportion of participants following the recommendations to isolate was higher among patients identified from the schools compared with those identified from the markets (31.9% versus 6.2%; *P* = 0.014). In addition, index patients identified from the participants were more likely to isolate than patients identified from the contacts, although the difference was not statistically significant (31.7% versus 23.9%; *P* = 0.319).

**Table 2:** Factors associated with positive test results

Characteristic	Number	*n* (%) Positive	Crude Prevalence Ratio	*P*-Value	Adjusted Prevalence Ratio	*P*-Value
Overall	10,942	185 (1.7)				
Type of respondent						
Participants	10,406	138 (1.3)	1		1	
Contacts	536	47 (8.8)	6.6 (4.8–9.1)	<0.001	6.2 (4.1–8.4)	<0.001
Place of testing/identification						
Market	1,545	15 (0.9)	1		1	
School	9,397	170 (1.8)	1.9 (1.1–3.2)	0.020	1.1 (0.6–2.1)	0.702
Sex*						
Male	5,331	60 (1.1)	1		1	
Female	5,075	78 (1.5)	1.4 (0.9–1.9)	0.068	1.1 (0.7–1.6)	0.698
H/O any chronic illness						
No	10,664	184 (1.7)	1			
Yes	278	1 (0.3)	0.21 (0.02–1.48)	0.117	0.3 (0.1–2.2)	0.238
Age (years)*						
<12	964	17 (1.8)	1		1	
12–18	4,476	78 (1.7)	0.9 (0.5–1.7)	0.964		
>18	4,966	43 (0.9)	0.5 (0.3–0.9)	0.012	1.6 (1.01–2.57)	0.045
Vaccination status						
None	3,900	92 (2.4)	1		1	
First dose only	4,619	60 (1.3)	0.6 (0.4–0.8)	<0.001	0.8 (0.5–1.3)	0.399
Fully vaccinated	2,320	33 (1.4)	0.6 (0.4–0.9)	0.012	1.0 (0.6–1.7)	0.975
Received a booster doze	103	0 (0.0)	0	N/A		
Any COVID-19 related symptom						
No	8,892	65 (0.7)	1		1	
Yes	2,050	120 (5.9)	8.0 (5.9–10.8)	<0.001	7.9 (5.5–11.3)	<0.001
H/O contact with suspect						
No	10,771	179 (1.7)	1		1	
Yes	171	6 (3.5)	2.1 (0.9–4.7)	0.067	1.6 (0.7–3.5)	0.254

COVID-19 = coronavirus disease 2019; H/O = History of.

* Data only available for participants.

### Number of infections averted and utility of the study.

To estimate the number of infections averted, we compared the test positivity estimates of the end-line surveys among participants in communities with regular or monthly testing to those from the control communities. As presented in Table 1, the baseline test positivity rates were comparable among the two groups (0.17% monthly testing group versus 0.18% control group; *P* = 0.944). In the end-line surveys, test positivity rates were higher than the baseline estimates for both the monthly testing communities and the control communities, which was possibly due to the ongoing COVID-19 wave at the time of the surveys (0.3% versus 0.17%; *P* = 0.432 for the monthly testing communities and 1.4% versus 0.18%; *P* <0.0001). However, in the end-line survey test, positivity rates were significantly lower in communities that had received monthly testing compared with control communities (5/1,678 [0.3%] versus 27/1,973 [1.4%]; protective efficacy = 78.2%; 95% CI 43.5–91.6%; *P* = 0.001; Table 3).

**Table 3:** Variation in test positivity rates between the intervention and control arms

Parameter	Control	Intervention	PR (95% CI)	*P*-Value
	*n*/*N* (%)	*n*/*N* (%)		
Baseline	3/1,661 (0.2)	3/1,750 (0.2)	0.95 (0.19–4.70)	0.944
Survey 2	N/A	39/1,596 (2.4)	—	—
Survey 3	N/A	66/1,748 (3.8)	—	—
Survey 4	27/1,973 (1.4)	5/1,678 (0.3)	0.22 (0.08–0.56)	0.001

### Factors associated with a positive Ag-RDT result.

At bivariate analysis, factors significantly associated with increased test positivity rates included 1) being a contact compared with being a volunteer (positivity rate [PR] = 6.6; 95% CI 4.8–9.1; *P* <0.001); 2) testing as part of the school surveys compared with testing as part of the market surveys (PR = 1.9; 95% CI 1.1–3.2; *P* = 0.020); and 3) having COVID-19 related symptoms compared with no symptoms (PR = 8.0; 95% CI 5.9–10.8; *P* <0.001). On the other hand, test positivity rates were significantly lower among adult participants (>18 years of age) compared with the younger children (PR = 0.5; 95% CI 0.3–0.9; *P* = 0.012) and participants who had received at least one vaccine dose compared with those who were not vaccinated (PR = 0.6; 95% CI 0.4–0.9; *P* = 0.007 for at least one dose of vaccine and PR = 0.6; 95% CI 0.4–0.9; *P* = 0.012 for the fully vaccinated). None of the participants who had received a booster dose tested positive on the SARS-CoV-2 Ag-RDT. In a multivariate analysis, factors that remained significantly associated with having positive Ag-RDT results included being a contact of a patient (PR = 6.2; 95% CI 4.1–8.4; *P* <0.001); being an adult (PR = 1.6; 95% CI 1.01–2.57; *P* = 0.045); and having COVID-19-related symptoms (PR = 7.9; 95% CI 5.5–11.3; *P* <0.001).

## DISCUSSION

In this operational research study, we evaluated the feasibility and utility of the SARS-CoV-2 Ag-RDT for the timely detection of and response to COVID-19 in schools and markets in two border districts of Uganda. Testing was successfully completed in 99.2% of the participants identified. The overall Ag-RDT test positivity rate for the study was 1.7%, with contacts, participants identified from the school surveys, and participants with symptoms having significantly higher test positivity rates. Receiving at least one dose of the COVID-19 vaccine was protective against COVID-19. The isolation of the patients identified was minimal, with less than one-third of the participants following the recommendation; however, even with the low number, communities with regular testing had significantly lower test positivity rates than the control communities.

Despite reductions in the SARS-CoV-2 disease burden, continued testing for COVID-19 is recommended to reduce morbidity and mortality through linkage to prompt care, to reduce onward transmission through the isolation of cases, and for disease surveillance to inform control.^21^ For COVID-19 testing to be effective in reducing transmission, the identification of cases must be extensive, with large-scale screening, and isolation must be ensured for all patients with and without symptoms.^22^^–^^24^ In this study, we demonstrate that identifying COVID-19 cases using SARS-CoV-2 Ag-RDTs and by tracing contacts is feasible in schools and markets in Uganda. It is important to note that the high levels of feasibility in testing may not depict the real-life setting, given that participants were compensated for their time. In addition, the isolation of the patients was inadequate, with only one in every three patients complying with COVID-19 isolation guidelines. Several reasons have been documented to affect adherence to isolation and quarantine guidelines, which have widely varied from sociocultural norms and the perceived prosocial character of isolation, the perceived benefits of quarantine, the perceived risk of disease outbreak, and trust in government.^25^ Self-isolation in resource-limited settings like Uganda is further challenged by inadequate housing, financial barriers to isolation in which most of the income is hand to mouth, and the inability to work from home.^26^ The study findings do not differ much from observations in other studies evaluating adherence to COVID-19 preventive measures in Uganda.^27^^,^^28^ In a study evaluating the adherence to four COVID-19 preventive measures (handwashing, wearing face masks, physical distancing, and coughing/sneezing hygiene) in Uganda, only 29% of the respondents were adherent to the guidelines.^27^ Another study evaluating compliance with COVID-19 prevention and control guidelines among supermarkets in Uganda showed that only 16.6% of the supermarkets complied with the guidelines.^28^

Contact tracing and the isolation of patients are common interventions for controlling infectious disease outbreaks. This strategy was also an effective control measure for smallpox and the severe acute respiratory syndrome epidemic in Hong Kong in 2003.^29^^,^^30^ Modeling studies have shown that case identification and isolation may reduce the incidence and transmission of COVID-19 infection.^24^^,^^31^ This is important because most SARS-CoV-2 transmission is attributed to individuals who are asymptomatic or pre-symptomatic.^32^ In this study, we demonstrate that despite the low levels of the isolation of patients, the communities that received regular or monthly testing had a 78.2% lower risk of having positive Ag-RDT results during the end-line survey compared with the control communities. We believe that the protection observed, despite limits in isolation, could be due to a number of reasons, including 1) volunteers who were worried about likely being infected were more likely to participate in the study and conducted the testing to allay their concerns and take precautions; 2) the few patients who isolated could have provided some protection to the population; 3) even when they failed to isolate, patients could have taken some additional protective measures, including the use of masks and social distancing, which we were unable to document in the study; and 4) the placebo effect due to the repeated visits to the intervention schools and markets by the study team could also have played a role. This is one of the few real-life studies that have demonstrated the utility of the wide-scale use of Ag-RDTs in reducing the community-level transmission of SARS-CoV-2.

In this study, contacts of index patients (school, workplace, and household contacts) were six times more likely to test positive for COVID-19 compared with the overall community. The most probable explanation for this is that when individuals live together or are in close physical contact for extended periods of time, the risk of droplet infection is increased, and this is worsened by the limited use of preventive measures in these environments. These findings add to the body of evidence that has shown an increased risk of acquiring COVID-19 infection among individuals living in close contact with patients.^33^^,^^34^ A meta-analysis of studies evaluating the secondary attack rates of COVID-19 in diverse contact settings showed that transmission differed significantly among contact settings, with the highest transmission occurring in households.^35^ It is important to note that the risk of testing positive among contacts was lower (and not significant) when the history of contact with a patient was used as the predictor variable, suggesting that depending on history may not be as sensitive in identifying the risk of transmission as the actual tracking of the cases.

Similar to previous reports, we observed that the test positivity rates were eight times higher when a participant had any COVID-19-related symptoms compared with when they were asymptomatic.^36^^,^^37^ We also observed that increasing age was marginally associated with higher test positivity rates. Although the explanation for this observation is still not clear, previous studies have associated this with more effective local control in younger SARS-CoV-2-exposed individuals.^36^^,^^38^^,^^39^

The study is not without limitations. Firstly, no data could be obtained on the acceptability of the approach as a public health intervention because all participants were compensated for their participation. It is important to note that the participation in testing could have been higher than what would be observed in real-life settings because of the compensation that was provided. Secondly, testing was conducted among volunteers, not a random sample of community members, which could have introduced selection bias. Thirdly, repeated testing was conducted monthly, but more frequent testing would have been more effective in demonstrating its utility. In addition, this study was conducted during a period of low COVID-19 prevalence in the country, which could explain the low positivity rates. Finally, a limited number of identified patients followed the isolation guidelines, which could undermine the effectiveness of the intervention.

## CONCLUSION

Despite these limitations, we conclude that repeated SARS-CoV-2 Ag-RDT testing is feasible and could be useful in reducing SARS-CoV-2 infections. However, isolation was limited, which may affect the magnitude of the impact of the intervention when rolled out on a large scale. Innovative strategies to increase the isolation of patients are needed to improve the utility of early testing for transmission reduction during epidemics.
